# Cognitive Strategy Training in Childhood-Onset Movement Disorders: Replication Across Therapists

**DOI:** 10.3389/fped.2020.600337

**Published:** 2021-01-21

**Authors:** Hortensia Gimeno, Helene J. Polatajko, Jean-Pierre Lin, Victoria Cornelius, Richard G. Brown

**Affiliations:** ^1^Complex Motor Disorders Service, Paediatric Neurosciences, Evelina London Children's Hospital, Guy's & St Thomas' NHS Foundation Trust, London, United Kingdom; ^2^Department of Psychology, King's College London, Institute of Psychiatry, Psychology and Neuroscience, London, United Kingdom; ^3^Department of Occupational Science and Occupational Therapy, University of Toronto, Toronto, ON, Canada; ^4^Imperial Clinical Trials Unit, Imperial College London, School of Public Health, London, United Kingdom; ^5^South London and Maudsley NHS Foundation Trust, London, United Kingdom

**Keywords:** single case experimental design, dystonia, cerebral palsy, rehabilitation, participation

## Abstract

**Objective:** To explore preliminary effectiveness of the Cognitive Orientation to daily Occupational Performance (CO-OP) Approach in improving outcomes in childhood-onset hyperkinetic movement disorders (HMDs) including dyskinetic cerebral palsy following deep brain stimulation (DBS) across UK clinical occupational therapists.

**Methods:** Randomized, multiple-baseline, Single Case Experimental Design N-of-1 trial with replications across participants. Five self-selected goals were identified: three goals were worked on during CO-OP and two goals were left untreated and used to assess skills transfer. Participants were between 6 and 21 years and had received DBS surgery with baseline Manual Ability Classification System (MACS) levels I–IV. Participants were randomized to typical or extended baseline (2 vs. 6 weeks), followed by 10 weekly individual CO-OP sessions. The primary outcome was functional performance measured by the Performance Quality Rating Scale-Individualized (PQRS-I), assessed before, during, and following treatment. Outcome assessors were blinded to baseline allocation, session number, and assessment time. A non-overlapping index, Tau-*U*, was used to measure effect size.

**Results:** Of the 12 participants recruited, 10 commenced and completed treatment. In total, 63% of trained goals improved with effect sizes 0.66–1.00 (“moderate” to “large” effect), seen for all children in at least one goal. Skills transfer was found in 37% of the untrained goals in six participants.

**Conclusions:** Cognitive strategy use improved participant-selected functional goals in childhood-onset HMD, more than just practice during baseline. Preliminary effectiveness is shown when the intervention is delivered in clinical practice by different therapists in routine clinical settings.

## Introduction

In children and young people with a hyperkinetic movement disorder (HMD) including dyskinetic cerebral palsy (CP) surgical treatments such as deep brain stimulation (DBS) can be effective in reducing severity of motor impairments (e.g., dystonia) (1, 2). However, children can be left with persistent functional problems in outperforming everyday tasks that are important to them and their families (3).

A recent systematic review of interventions for CP indicated strong evidence that surgical interventions, such as intrathecal baclofen infusion pump or selective dorsal rhizotomy, and medical interventions, such as botulinum toxin injections, reduce tone in children with spastic CP (4). However, it is the combination of these interventions with adjunct rehabilitation such as strength training or occupational therapy that yield effective results in improving motor and/or functional outcomes. However, there is scant evidence to guide rehabilitation practice following interventions such as DBS in children and young people (4) and only a few small studies in adult-onset dystonia (5, 6). Even in specialist centers, number of cases are relatively small, and the heterogeneity of these disorders in terms of etiology, motor severity, and non-motor factors make the planning and implementation of large scale randomized controlled trials (RCTs) challenging.

Nevertheless, rehabilitation approaches are available but, to date, lack robust evidence for their feasibility and efficacy. We have recently reported a proof-of-concept efficacy study (7) of the Cognitive Orientation to daily Occupational Performance (CO-OP) (8) used with children and young people with HMD and DBS in place. CO-OP is an individualized, client-centered approach that uses personalized strategies to achieve client-chosen functional goals. The results provided preliminary evidence to support the feasibility, acceptability, and potential efficacy, evaluated using single-case experimental design replications (7). In that study, treatment was delivered by a single experienced occupational therapist in a specialist pediatric movement disorder service. It is important to explore if these findings can be replicated in routine settings and implemented by local therapists.

Therapist effects, including training and experience, can be a fundamental variable in studies investigating any rehabilitation intervention effectiveness (9–11). Single-case experimental design methodology provides the opportunity, with limited numbers, to simultaneously investigate treatment efficacy and therapist effects (12, 13).

This study aimed to explore whether the results from a previous single-case experimental design proof-of-concept (efficacy) study (7, 14) could be replicated by other therapists (adherence to protocol) based in non-specialized services (effectiveness) (15). Further, the study sought to assess the impact of practice by using an extended baseline with repeated assessment of participant-selected goals prior to commencing treatment.

## Methods

The study evaluated the CO-OP approach, as an adjunct to DBS, delivered by non-specialist occupational therapists with a range of clinical experience, to children and young people with HMD. The full trial protocol is available (15) and was implemented with minor changes. Single-case experimental design methodology followed the Single Case Reporting Guideline in Behavioral Interventions (SCRIBE) (12).

### Research Question

Does the CO-OP approach improve outcomes for children and young people with HMD on participant-selected goals when undertaken in a non-specialized clinical setting?

### Standard Protocol Approvals, Registrations, and Patient Consents

Ethical approval was obtained by the NHS Health Research Authority (Oxford A Research Ethics Committee, 14/SC/1159). The trial was registered (ISRCTN57997252). Written informed consent was obtained from all parents and from participants over 12 years of age. Assent was obtained from younger children. Written permission was obtained in all cases.

### Design

This study used a randomized, multiple-baseline, N-of-1 design with replications across six therapists (N-of-1 with five replications) in 12 children and young people (N-of-1 trial with 11 replications) (NB: number achieved was N-of-1 with 9 replications across participants).

A consecutive series of multiple baseline N-of-1 trials was completed using concealed randomization to allocate length of baseline (2 vs. 6 weeks). The extended baseline permitted an examination of whether repeated baselining (practicing the goals without CO-OP input) and DBS only has an impact on skill improvement.

### Sample Size

In an N-of-1 design with replications across participants, sample (series) size is not based on the power to test group effects using inferential statistics. Instead, each separate trial examines change over time within an individual, thus allowing us to determine whether treatment is effective for each individual using predefined quantitative and qualitative criteria. In N-of-1 studies, the number of replications (participants recruited) chosen after the first case is often based on pragmatic grounds related to the known heterogeneity of the clinical sample. The number of measurements within each time period is also based on pragmatic factors such as length of treatment and the likely variability in outcome. It is recommended that an initial N-of-1 trial plus three replications are necessary as a minimum to explore efficacy of an intervention but five replications is better (16). Similarly, it is recommended that at least three assessment points are measured as a baseline before the intervention is introduced (16) but preferably five (17). A sample size of 10 was judged to be sufficient to provide information on whether the intervention produced meaningful clinical change for individuals, to examine the direction of “average treatment effect” across individuals, and to provide estimates of the within- and between-subject variability of outcomes in this population. This sample size will also allow us to obtain information on ease of recruitment and adherence to protocol that might inform any future clinical trial. As standard in N-of-1 trial, this number of participants will allow for statistical analysis of effect size of the intervention relative to baseline.

### Participants

The study included children and young people with HMD who had previously undergone DBS (or where surgery was scheduled) at the complex motor disorders service (CMDS) database at Evelina London Children's Hospital, UK, that met the inclusion criteria from information in their medical records (*n* = 27). The 10 patients that had participated in the previous study were not eligible. Full inclusion criteria for the study has been reported elsewhere (7): (a) diagnosis of pediatric HMD other than neurodegenerative conditions; (b) sufficient receptive and expressive communication ability to follow simple instructions and engagement with treatment; (c) age 6 to 21 years; (d) Manual Abilities Classification System (MACS) levels I–IV; (e) emerging skills in self-care; (f) ability to mobilize independently; (g) cognitive ability of 6 years of age or IQ above 70; and (h) DBS electrodes *in situ* and without signs of infection.

**Figure 1:**
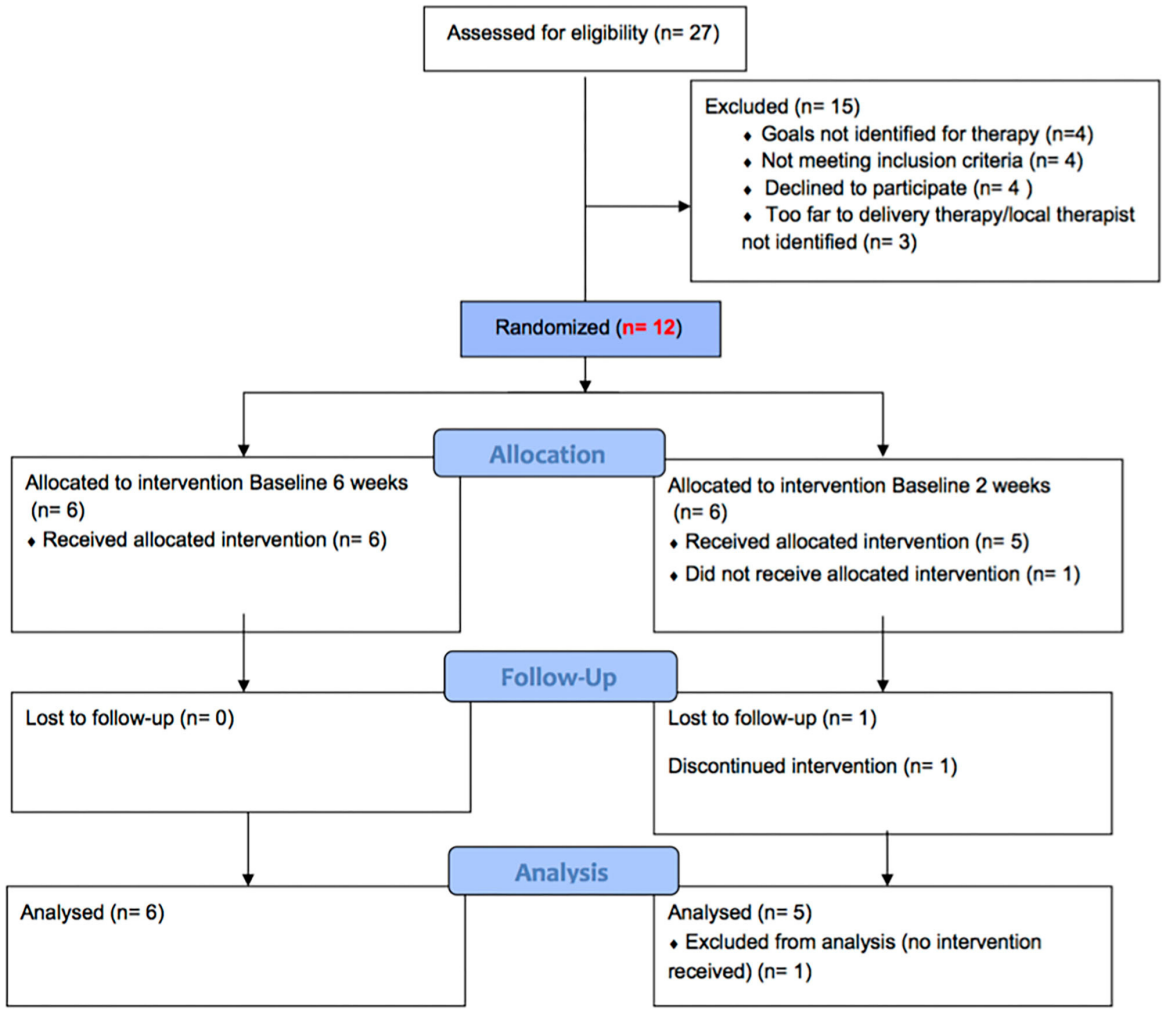
Recruitment diagram shows the total number of participants approached.

Twelve participants were recruited and admitted to the study sequentially. Further information about eligibility criteria and recruitment procedure are outlined in the study protocol (14, 15). Figure 1 describes the recruitment process and randomization to two arms and reasons for exclusion. Details of the participants are provided in Table 1.

**Table 1:** Participant demographic and clinical characteristics (*N* = 12).

**Child**	**Therapist**	**Age**	**Gender**	**Randomization group**	**Diagnosis**	**Etiology**	**Phenotype**	**DBS duration**	**GMFCS**	**MACS**
1	1	11 y 8 m	Female	2 weeks	Dyskinetic CP	Acquired (secondary to Kernicterus)	Dystonia	6 m	II	IV
2	2	16 y 11 m	Female	2 weeks	Dyskinetic CP secondary to maternal ruptured uterus	Acquired (secondary)	Dystonia and choreoathetosis	4 y	III	IV
3^*^	Dropped out	19 y 4 m	Female	6 weeks	Early onset generalized dystonia. DYT-TOR1A (DYT-1)	Inherited (Primary)	Dystonia	2 y 6 m	III	II
4	3	8 y 6 m	Female	6 weeks	Stroke	Acquired (secondary)	Dystonia	6 m	II	II
5	4	19 y 10 m	Female	2 weeks	Childhood-onset progressive dystonia. KMT2B (DYT28)	Inherited (Primary)	Dystonia	9 m	II	III
6	5	16 y 7 m	Male	2 weeks	Dyskinetic CP	Acquired (secondary to HIE)	Dystonia and choreoathetosis	6 m	II	II
7^**^	Not eligible	14 y	Female	6 weeks	Dopa Responsive Dystonia	Inherited (Primary)	Dystonia	–	III	II
8	4	9 y 8 m	Female	6 weeks	Myoclonus dystonia. DYT-SGCE (DYT-11)	Inherited (Primary)	Dystonia and myoclonus	2 m	I	II
9	6	9 y 3 m	Male	6 weeks	GA-1	Acquired (secondary)	Dystonia and chorea	3 y	III	IV
10	4	18 y 11 m	Male	2 weeks	Stroke	Acquired (secondary)	Dystonia	6 m	II	II
11	4	17 y 4 m	Male	2 weeks	Dyskinetic CP secondary to maternal placental abruption	Acquired (secondary)	Dystonia and chorea	15 m	II	II
12	4	15 y 9 m	Female	6 weeks	Dyskinetic CP	Acquired (secondary to HIE)	Dystonia and myoclonus	4 y 6 m	I	II

*Child number reflects the order of recruitment*.

* *Case 3 withdrew for personal reasons after baseline*.

** *Case 7 was no longer eligible for the study at the start of baseline assessment*.

*DBS, Deep Brain Stimulation; GMFCS, Gross Motor Function Classification System; MACS, Manual Ability Classification System; CP, Cerebral Palsy; HIE, Hypoxic Ischemic Encephalopathy; GA, Glutaric Aciduria type 1; y, year; m, month*.

*Table 1 shows demographic characteristics for all cases recruited to this study with cases organized firstly by those allocated to 2 weeks baseline followed by the participants allocated for 6 weeks baseline. Child number reflects the order of recruitment. Diagnosis, etiology, and phenotype are provided as well as DBS duration, DBS, and MACS levels*.

### Therapists

The Evelina Children's Hospital is a tertiary treatment center providing advanced interventions for children with complex motor disorders. Children are referred from across the UK. For this trial, 19 therapists were initially contacted in centers close to the homes of the 27 potential participants, and 3 additional therapists were approached via special interest group networks. Seven agreed to take part in the study prior to enrolment of the first participant, with six eventually matched to one or more patient participants. Details of the therapists are provided in Supplementary Information 1.

### Intervention Description

CO-OP intervention comprised 10 treatment sessions of up to 1 h each, delivered weekly at the participant's home. The frequency of intervention sessions was negotiated between the treating therapist and the participant and family. All therapists had attended CO-OP training workshops (2–3 days) led by CO-OP Academy certified instructors. Ongoing clinical supervision was provided by investigator HG. For more information, please see study protocols (14, 15).

### Primary Outcome

Performance change in self (participant)-selected goals was measured using the Performance Quality Rating Scale—individualized (PQRS-i) (7). This is a 10-point observational scale, with 1 representing “cannot do the task at all” and 10 representing “does task very well.” The PQRS-i does not penalize the child or young person for how the task is performed or whether dystonia is present or not. PQRS-i is a scale based on observation of the behavior (behavior defined as the task at hand). The child is asked to perform the chosen goals, in their natural environment (i.e., their home), using utensils and materials familiar to them. Performance was videoed by the primary investigator. Performance scores were obtained by rating video recordings of all baselines and post-intervention sessions and a sample of eighteen 5-min randomly selected video segments of the intervention sessions. The videos were randomly presented in a non-chronological order and rated by a trained independent, blinded PQRS-i rater. For more information about how the performance clips for intervention were selected, please refer to the study protocol (14).

Assessments of outcomes were completed several times at each study phase (baseline, during treatment, and end of treatment). Five task-based goals were self-identified by each participant with the aid of the Canadian Occupational Performance Measure (COPM) (18) (data not reported here). Three of the goals were addressed in the CO-OP intervention (trained goals) and two, which were not addressed in the sessions with the therapist, were used to assess generalization and transfer (untrained goals). The participants chose which goals they wished to work on in therapy.

### Analysis

Outcomes and significance of change were analyzed and evaluated using a set of complementary approaches following SCRIBE guidelines. Improvement is also summarized for the group in terms of number of goals improved.

#### Visual Analysis

Changes in means, levels, trends, variability, latency, and consistency were evaluated using visual graph analysis (19–21). PQRS-i data were graphed with separate data points in each phase (baseline, intervention, and post-intervention).

#### Quantitative Statistical Analysis of Performance Change Between Baseline and Post-intervention

Serial dependency at baseline was firstly calculated using auto-correlation (AC) so that the most appropriate analysis approach could be chosen, which were (i) differences in individual means and 95% confidence interval for overall change pre- and post-*t-*test; (ii) regression, initially fitting a naïve linear regression model using ordinary least squares (OLS) for reference only (22, 23); and (iii) effect size calculated using non-overlapping index of the Kendall's Tau for non-overlap with baseline trend control (Tau-*U*) (24) taking into consideration baseline AC in the calculation. Effect size is considered “large” (“very effective intervention”) when Tau-*U* is ≥0.93, “moderate” (“effective intervention”) for values 0.66 to 0.92, and “weak” when values are ≤0.65.

#### Clinically Significant Change Between Baseline and Post-intervention

A change of at least 2 points on the PQRS-i was used to indicate clinical significance in order to match other similar scales such as the COPM (18). Differences between mean scores from pre- and post- were calculated for each individual for each goal.

Quantitative statistical analysis of performance change between CO-OP and treatment as usual (DBS and practice of the goals): Changes in performance across the extended baseline (6 weeks with at least 18 data points) was specifically evaluated. Differences between individual goal means and 95% confidence interval for change of first session and sixth session of baseline (*t-*test) were calculated for those participants randomized to 6 weeks baseline length.

#### Analysis of Results in Relation to Therapist-Related Fidelity to Treatment

Fidelity to treatment was evaluated by reviewing randomly selected video-recorded treatment sessions by a CO-OP expert external to the study team using the CO-OP Academy fidelity checklist (25). The randomly selected treatment session per therapist was evaluated fully by the external expert. Therapist factors (training and years of experience) and the fidelity to treatment were explored for their possible impact on clinical outcome.

### Adherence to CO-OP Protocol

Fidelity to treatment was evaluated by reviewing randomly selected video-recorded treatment sessions by a CO-OP expert external to the study team using the CO-OP Academy fidelity checklist (25). The randomly selected treatment session per therapist was evaluated fully by the external expert.

## Results

The participants (eight female, four male) ranged in age from 8 years and 6 months to 19 years and 10 months, with a range of primary diagnoses, etiologies, and clinical phenotypes (Table 1). Duration since DBS ranged from 6 months to 4 years 6 months. Of the 12 participants, 1 was excluded before functional assessments and intervention commenced as scheduled surgery was canceled, and one withdrew following baseline assessment. Of the remainder, all completed the planned 10 CO-OP sessions. For completeness, details of all 12 participants are provided in the results tables. A summary of statistical and clinical significance is represented in Table 2 and more detail is shown in Table 3 with statistical parameters and results.

**Table 2:** Summary of outcomes (mean change, effect size, clinical significance) with three separate analyses of the individual goals per participant.

**Child**	**Trained goals**	***t*-test difference in means**	**Effect size (Tau-*U*)**	**CSC**	**Untrained goals**	***t*-test difference in means**	**Effect size (Tau-*U*)**	**CSC**
1	G1: Putting T-shirt	No change	Weak	Improved	G4: Eating with a spoon |	Improved	Moderate	Improved
	G2: Drinking from open cup	Improved	Moderate	Improved	G5: Applying lip balm	No change	Weak	No change
	G3: Pouring water	Improved	Strong	Improved				
2	G1: Drinking	No change	Weak	No change	G4: Eating crisps	Worse	Deteriorates mod	Worse
	G2: Brushing teeth	Improved	Strong	Improved	G5: Doing buttons	No change	Weak	No change
	G3: Pouring water	Improved	Moderate	Improved				
4	G1: Doing zips	Improved	Moderate	Improved	G4: Opening a snack	No change	Weak	Improved
	G2: Doing buttons	No change	Weak	Improved	G5: Putting socks on	Worse	Deteriorates mod	Worse
	G3: Putting shoes on	No change	Weak	No change				
5	G1: Applying mascara	Improved	Moderate	Improved	G4. Applying lipstick	Improved	Moderate	No change
	G2: Making a ham sandwich	Improved	Strong	Improved	G5: Brushing teeth	No change	Weak	No change
	G3: Carrying a cup of tea	Improved	Moderate	Improved				
6	G1: Buttering and cutting bread	Improved	Strong	Improved	G4. Writing signature on small window in paper	Improved	Moderate	Improved
	G2: Cooking in oven and taking out dish	No change	Weak	No change	G5: Cutting an apple	Improved	Strong	Improved
	G3: Cooking pasta	Improved	Moderate	Improved				
8	G1: Drinking without spilling	No change	Weak	No change	G4. Pouring water	No change	Weak	Improved
	G2: Handwriting	Improved	Moderate	Improved	G5: Carrying water	No change	Weak	Improved
	G3: Stirring food	Improved	Strong	Improved				
9	G1: Eating with a spoon	No change	Weak	Improved	G4. Brushing teeth	Improved	Moderate	No change
	G2: Drinking without spilling	Improved	Moderate	Improved	G5: Putting a t-shirt on	No change	Weak	No change
	G3: Riding a bike	No change	Weak	No change				
10	G1: Carrying a cup of tea	No change	Weak	No change	G4. Putting sheet on plastic folder	No change	Weak	No change
	G2: Cutting fingernails	Improved	Strong	Improved	G5: Opening tin of tomatoes	Improved	Moderate	Improved
	G3: Doing shoelaces	Improved	Strong	Improved				
11	G1: Cutting bread	Improved	Strong	Improved	G4. Leg elevation exercise	No change	Weak	No change
	G2: Putting socks on	Improved	Strong	Improved	G5: not set			
	G3: External rotation hip exercise	Improved	Strong	Improved				
12	G1: Applying mascara	Improved	Strong	Improved	G4. Carrying water	Improved	Strong	Improved
	G2: Drinking from a glass	No change	Weak	No change	G5: Eating with a spoon	No change	Weak	No change
	G3: Eating with knife and fork	No change	Weak	No change				

*G, Goal; CSC, clinically significance change; NT, Not tested*.

**Table 3:** Results including statistical at-test analysis and changes in slope with negative *T*-test indicating improvement in change pre-post scores whilst positive *t*-test scores indicate negative trend.

**Child number (goal)**		**Weeks**	**Baseline**	**Post Rx**	**Post**	**Trend**	***T*****-test post-pre-**		**Slope beta (p)**
	**AC**	**Length**	**Mean (SD)**	**Mean (SD)**	**PQRS 2p change**		**Mean shift (95% CI)**	***P***	
**TRAINED GOALS**									
1 (1)	1.80	2	3.17 (2.40)	5.33 (0.52)	Yes	↑	−2.17 (−4.68, 0.35)	0.078	−0.454 (0.103)
1 (2)	Constant	2	1.0 (0.00)	2.00 (0.89)	No	None	−1.00 (−1.94, −0.06)	0.041	0.187 (0.314)
1 (3)	Constant	2	1.0 (0.00)	5.00 (1.79)	Yes	↑	−4.00 (−5.88, −2.12)	0.003	0.553 (0.001)
2 (1)	2.429	2	2.00 (1.26)	1.60 (0.89)	No	None	0.40 (−1.08, 1.88)	0.56	−0.077 (0.676)
2 (2)	2.177	2	4.00 (1.15)	6.00 (0.00)	Yes	None	−2.00 (−3.84, −0.16)	0.041	0.339 (0.156)
2 (3)	0.934	2	2.17 (0.75)	4.67 (1.86)	Yes	↑	−2.50 (−4.46, −0.54)	0.020	0.612 (0.015)
5 (1)	1.989	2	4.86 (1.07)	7.17 (1.17)	Yes	↑	−2.31 (−3.70, −0.92)	0.004	0.605 (0.0005)
5 (2)	2.252	2	2.33 (0.52)	5.50 (0.55)	Yes	↑	−3.17 (−3.85, −2.48)	<0.001	0.446 (0.029)
5 (3)	1.480	2	4.00 (1.00)	6.83 (2.14)	Yes	↑	−2.83 (−5.09, −0.58)	0.021	0.434 (0.039)
6 (1)	3.000	2	2.67 (1.15)	6.83 (1.17)	Yes	↑	−4.17 (−6.41, −1.92)	0.006	0.676 (0.006
6 (2)	0.0000	2	9.50 (0.71)	9.50 (0.84)	No	None	0 (−2.53, −2.53)	1	0.260 (0.350)
6 (3)	3.000	2	4.33 (1.53)	7.50 (2.43)	Yes	↑	−3.17 (−6.38, 0.05)	0.053	0.330 (0.155)
10 (1)	2.853	2	9.0 (0.63)	9.00 (1.67)	No	None	0 (−1.76, 1.76)	1	0.000 (1.000)
10 (2)	2.449	2	1.33 (0.52)	9.67 (0.52)	Yes	↑	−8.33 (−9.00, −7.67)	<0.001	0.625 (0.000)
10 (3)	2.498	2	1.17 (0.41)	10.00 (0.00)	Yes	↑	−8.33 (−9.26, −8.40)	<0.001	0.888 (0.000)
11 (1)	1.504	2	3.0 (0.63)	6.67 (2.25)	Yes	↑	−3.67 (−6.02, −1.31)	0.009	0.382 (0.021)
11 (2)	2.578	2	6.0 (1.67)	10.00 (0.00)	Yes	↑	−4.00 (−5.76, −2.24)	0.002	0.873 (0.000)
11 (3)	2.547	2	1.14 (0.38)	5.00 (0.00)	Yes	↑	−3.86 (−4.21, −3.51)	<0.001	0.873 (0.000)
3 (1)	1.697	6	4.81 (1.21)	No Rx		None	Dropped out		
3 (2)	2.379	6	6.76 (0.89)	No Rx		None	Dropped out		
3 (3)	1.216	6	5.29 (2.10)	No Rx		None	Dropped out		
4 (1)	2.120	6	1.06 (0.24)	7.00 (4.65)	Yes	↑	−5.94 (−10.82, −1.06)	0.026	0.626 (0.0000)
4 (2)	1.005	6	2.12 (1.58)	4.60 (3.36)	Yes	↑	−2.48 (−6.60, 1.63)	0.176	0.431 (0.019)
4 (3)	Constant	6	1.0 (0.00)	2.80 (3.49)	No		−1.80 (−6.14, 2.54)	0.313	0.262 (0.141)
8 (1)	2.226	6	4.25 (2.02)	4.33 (3.87)	No	None	−0.08 (−3.63, 3.46)	0.956	0.191 (0.296)
8 (2)	2.018	6	4.05 (1.27)	6.83 (0.75)	Yes	↑	−2.78 (−3.68, −1.88)	<0.001	0.585 (0.001)
8 (3)	1.901	6	3.44 (2.55)	9.00 (2.00)	Yes	↑	−5.56 (−7.10, −4.02)	<0.001	0.669 (0.000)
9 (1)	1.875	6	4.61 (1.85)	5.75 (1.16)	No	None	−1.14 (−2.39, 0.11)	0.072	0.139 (0.337)
9 (2)	1.686	6	1.94 (1.39)	4.50 (1.05)	Yes	↑	−2.56 (−3.74, −1.37)	<0.001	0.564 (0.001)
9 (3)	Constant	6	1.0 (0.00)	1.00 (0.00)	No	None	−4.00 (−6.51, −1.49)	0.009	0.210 (0.188)
12 (1)	1.981	6	3.0 (1.93)	10.00 (0.00)	Yes	↑	−7.00 (−8.07, −5.93)	<0.001	0.837 (0.000)
12 (2)	1.404	6	1.94 (1.89)	1.67 (0.58)	No	None	0.28 (−0.94, 1.50)	0.628	0.194 (0.232)
12 (3)	2.263	6	4.06 (2.43)	3.00 (1.00)	No	↓	1.06 (−0.89, 3.01)	0.243	0.299 (0.046)
			**Baseline**	**Post Rx**	**Post**	**Trend**	***T*****-test pre–post**		**Slope beta (p)**
	**AC**		**Mean (SD)**	**Mean (SD)**	**2p**		**Mean shift (95% CI)**	***P***	
**UNTRAINED GOALS**									
1 (4)	1.258	2	5.00 (1.94)	8.50 (1.05)	Yes	↑	−3.50 (−5.11, −1.89)	<0.001	0.733 (0.001)
1 (5)	1.361	2	2.50 (1.64)	3.50 (1.05)	No	None	−1.00 (−2.81, 0.82)	0.242	0.369 (0.237)
2 (4)	2.394	2	4.00 (1.67)	2.17 (0.41)	No	↓	1.83 (0.08, 3.58)	0.043	−0.636 (0.026)
2 (5)	Constant	2	1.0 (0.00)	1.0 (0.00)	No	None	−0.90 (−9.14, 7.34)	0.664	Constant
5 (4)	2.107	2	2.83 (0.75)	4.71 (0.95)	No	↑	−1.88 (−2.92, −0.84)	0.002	0.762 (0.002)
5 (5)	1.654	2	4.67 (1.03)	5.60 (0.55)	No	None	−0.93 (−2.06, 0.20)	0.093	0.516 (0.104)
6 (4)	3.400	2	4.00 (1.83)	7.50 (2.35)	Yes	↑	−3.50 (−6.57, −0.43)	0.031	0.663 (0.037)
6 (5)	3.000	2	2.67 (0.58)	7.17 (1.83)	Yes	↑	−4.50 (−6.47, −2.53)	0.001	0.836 (0.005)
10 (4)	2.842	2	7.17 (1.47)	8.33 (1.86)	No	None	−1.17 (−3.34, 1.01)	0.258	0.356 (0.256)
10 (5)	1.249	2	6.33 (1.51)	8.67 (0.52)	Yes	↑	−2.33 (−3.91, −0.75)	0.011	0.750 (0.005)
11 (4)	1.586	2	4.29 (1.50)	5.00 (1.41)	No	None	−0.71 (−2.41, 0.98)	0.377	0.253 (0.377)
11 (5)	Not set	2	N/A	N/A	N/A	N/A	N/A	N/A	N/A
3 (4)	1.780	6	2.38 (2.66)	No Rx		None	Dropped out		
3 (5)	1.987	6	3.43 (1.55)	No Rx		None	Dropped out		
4 (4)	2.304	6	5.25 (4.43)	8.25 (3.06)	Yes	↑	−3.00 (−6.24, 0.24)	0.067	−0.343 (0.101)
4 (5)	1.887	6	7.54 (1.76)	4.25 (2.50)	No	↓	3.29 (−0.45, 7.03)	0.071	−0.609 (0.009)
8 (4)	1.853	6	5.44 (2.20)	8.00 (3.16)	Yes	↑	−2.56 (−5.88, 0.77)	0.111	0.426 (0.038)
8 (5)	1.749	6	6.43 (3.74)	8.67 (3.27)	Yes	None	−2.23 (−5.74, 1.28)	0.183	0.248 (0.194)
9 (4)	1.903	6	2.11 (0.68)	4.00 (1.26)	No	↑	−1.89 (−3.21, −0.57)	0.013	0.710 (0.000)
9 (5)	0.732	6	5.06 (1.80)	6.00 (2.00)	No	None	−0.94 (−3.07, 1.82)	0.335	0.225 (0.289)
12 (4)	0.960	6	1.41 (1.00)	6.33 (1.53)	Yes	↑	−4.92 (−8.39, −1.46)	0.024	0.865 (0.000)
12 (5)	0.726	6	3.88 (2.12)	3.33 (0.58)	No	None	0.39 (−0.77, 1.87)	0.386	−0.102 (0.667)

*AC, Auto-correlation; SD, Standard Deviation; CSC, Clinically Significance Change; CI, Confidence Interval; NT, non-tested; ↑, Ascendant; ↓, Descendent*.

*Table 3 shows three separate analysis of the individual goals per participant. Negative T-test indicates improvement in change pre–post scores whilst positive t-test scores indicates negative trend*.

### Outcome

#### Visual Analysis

**Figure 2:**
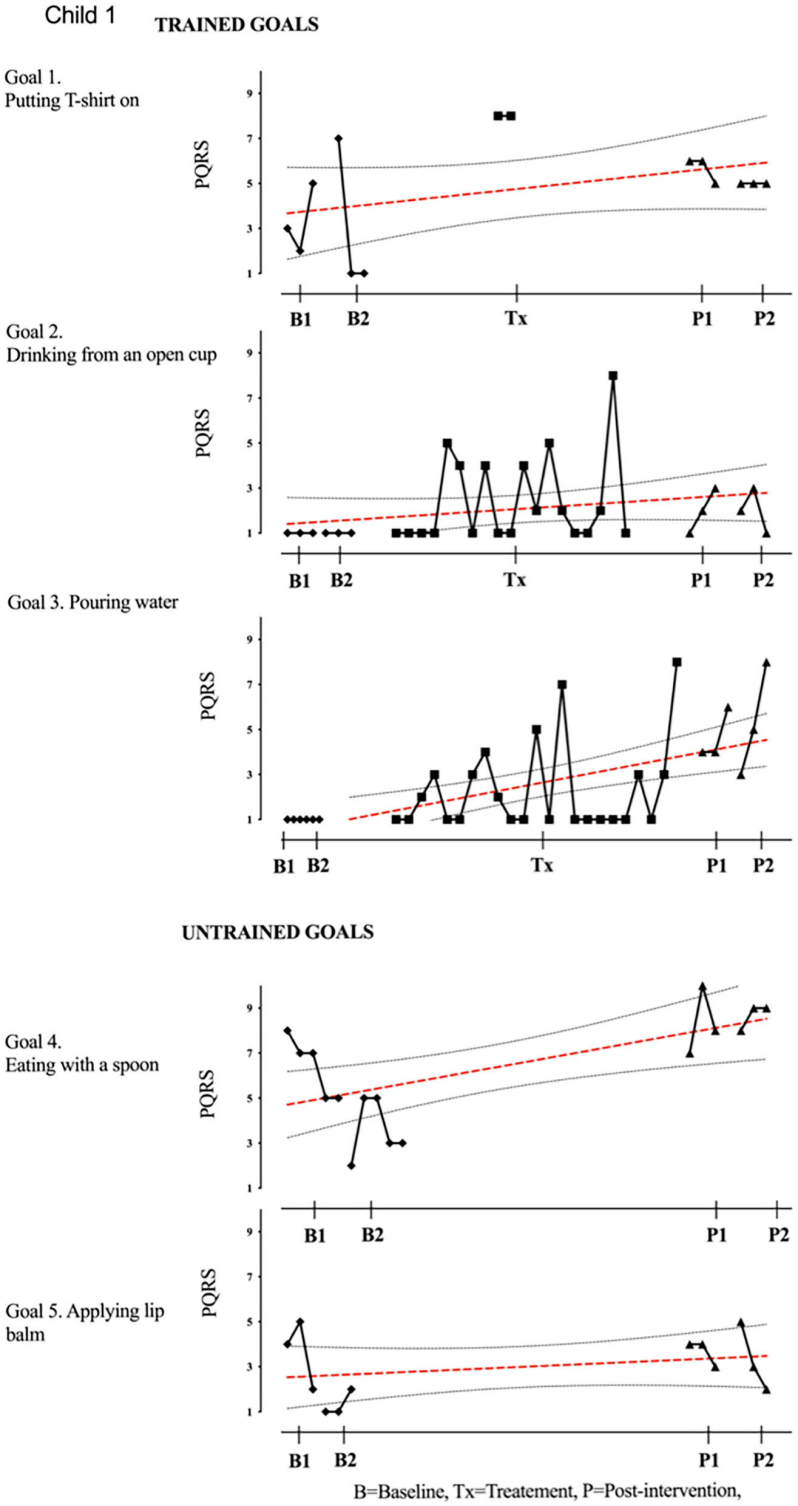
Participant 1, PQRS-i scores (y-axis) for Gl-5 for each trial phase (x-axis). For participant 1, the graph indicates a significant improvement in goals 2–4 with stable baseline on goals 2 and 3 (drinking from an open cup and pouring water) and change in slope observed during the treatment session. Even though improved, variability of performance was observed at the post-intervention phase. The visual data provides evidence on means, levels, trends, variability, latency, and consistency across the different phases of the study for each participant and for each individual goal. Also shown is the OLS regression line and 95% confidence range.

**Figure 3:**
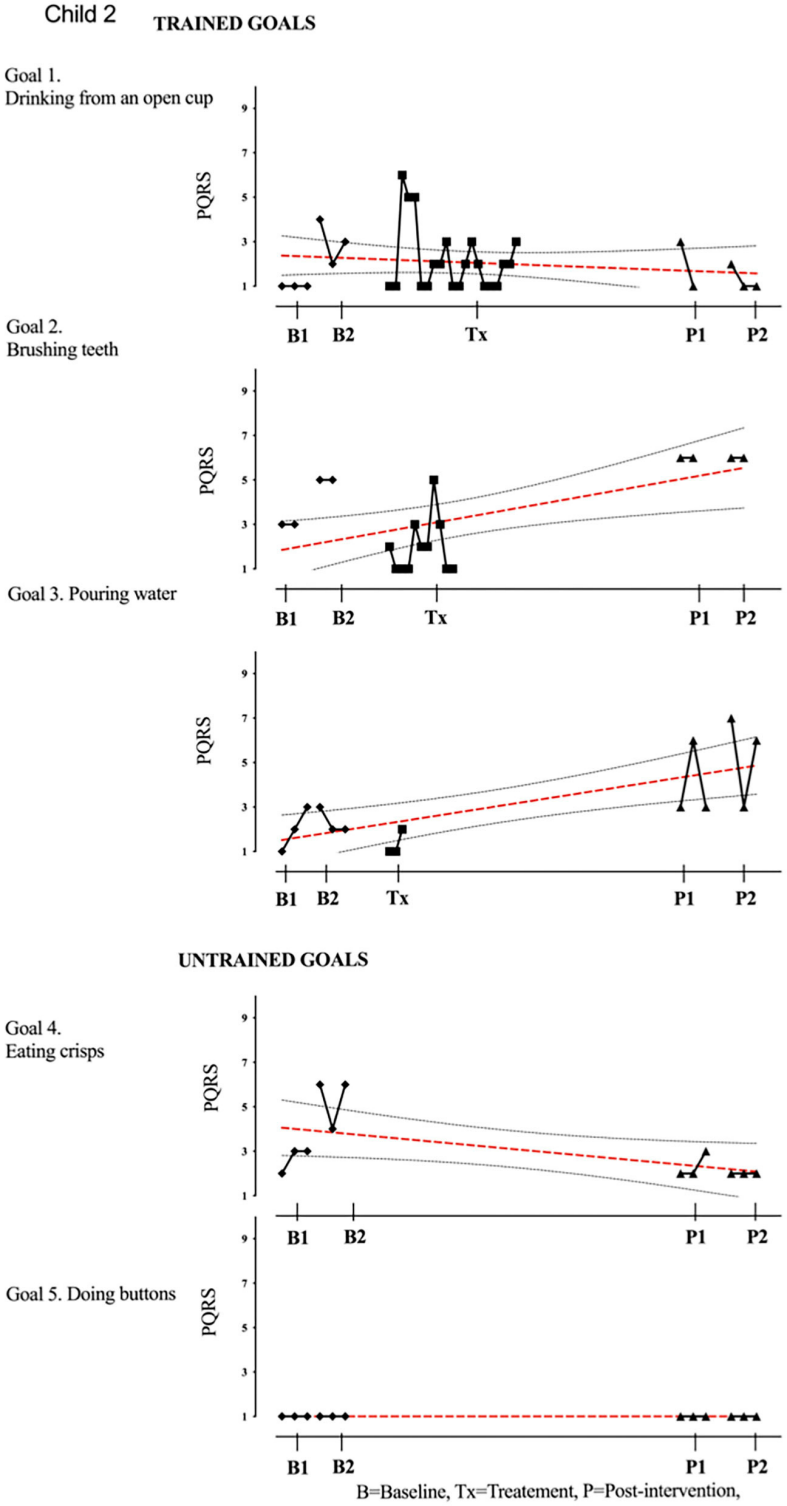
Child 2 PQRS-i scores (y-axis) for Gl-5 for each trial phase (x-axis). Supplementary Information 3. Child 2. Results with OLS regression line superimposed.

**Figure 4:**
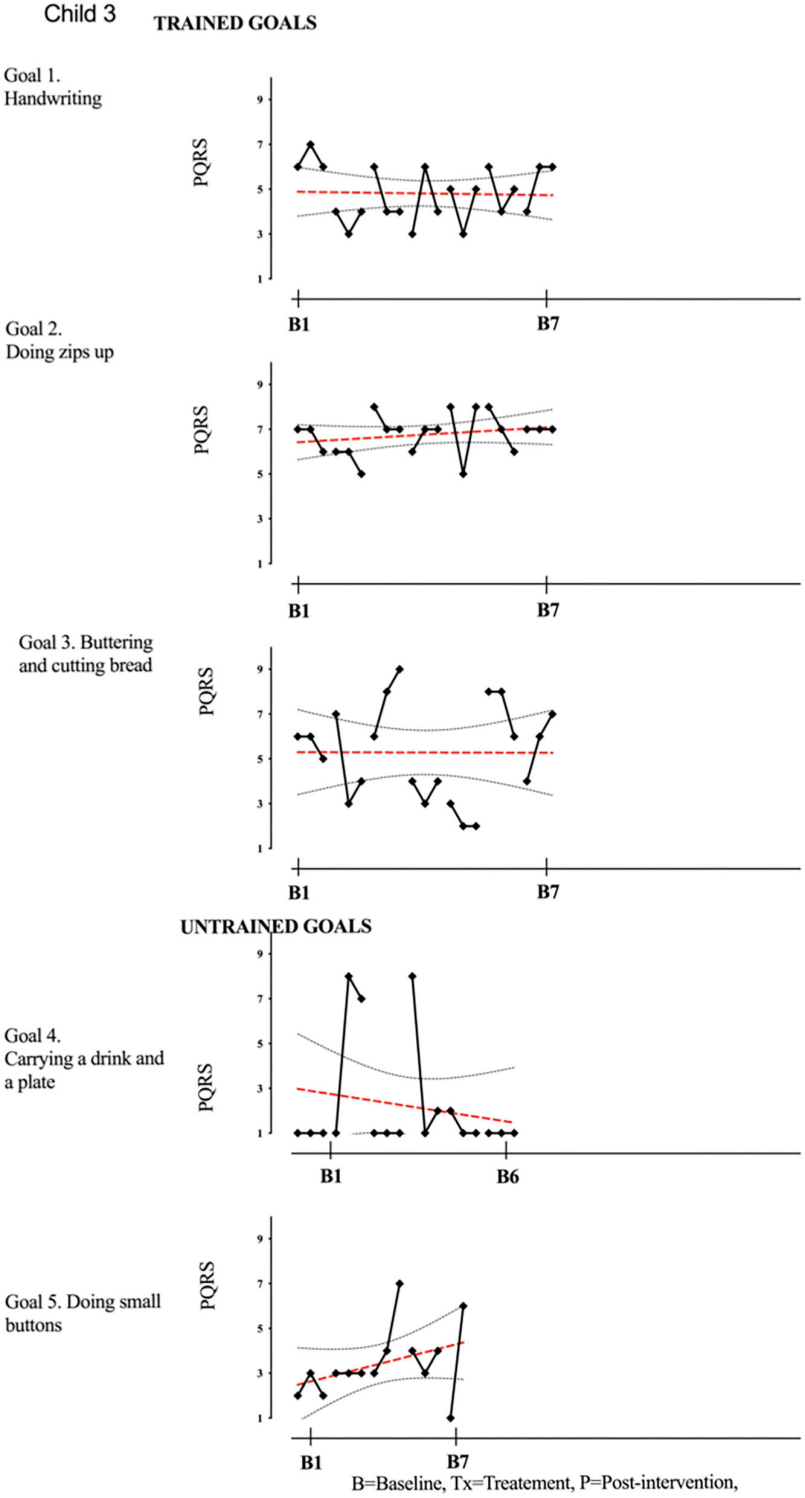
Child 3 PQRS-i scores (y-axis) for Gl-5 for each trial phase (x-axis). Results with OLS regression line superimposed.

**Figure 5:**
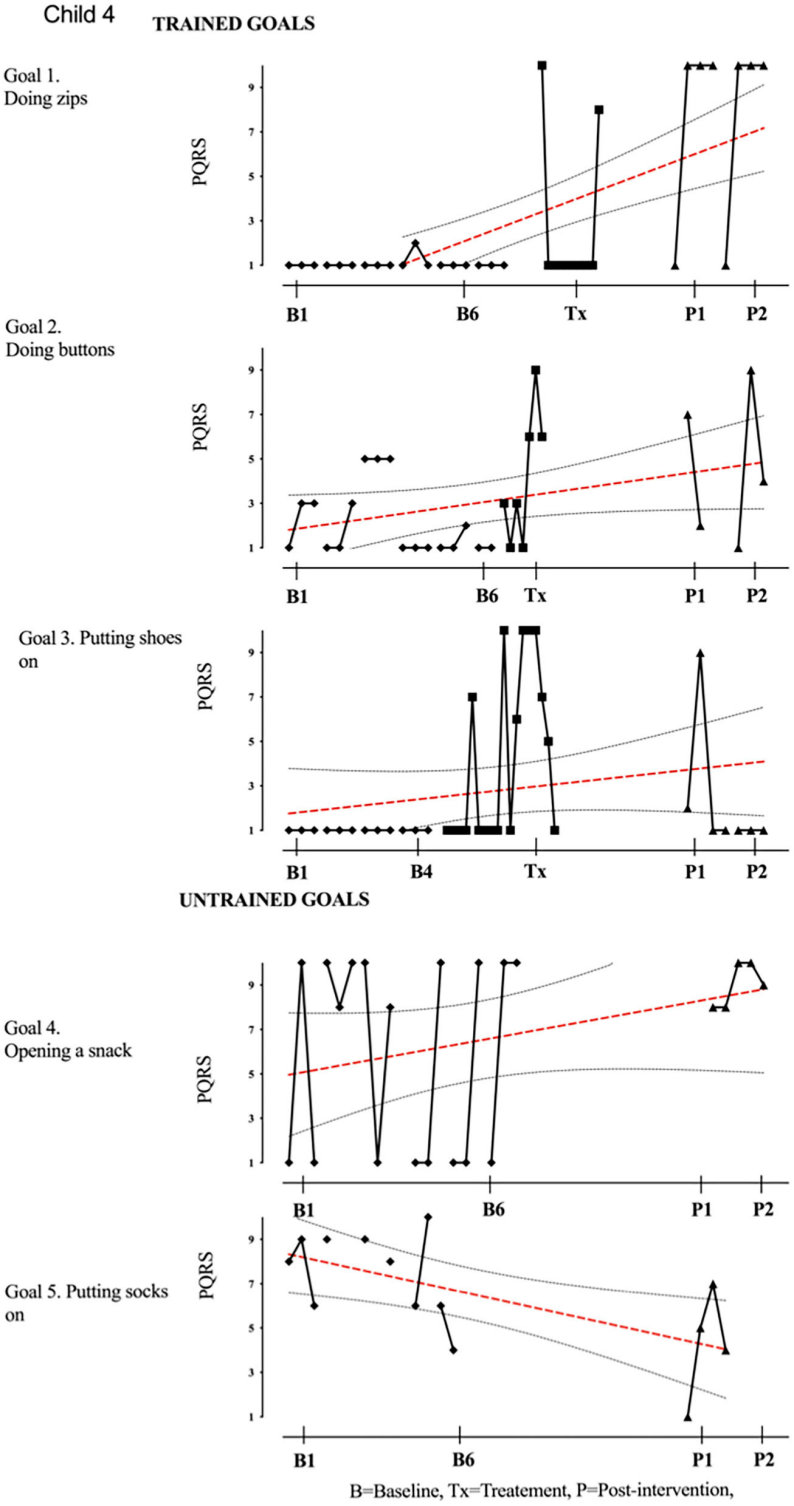
Child 4 PQRS-i scores (y-axis) for Gl-5 for each trial phase (x-axis). Results with OLS regression line superimposed.

**Figure 6:**
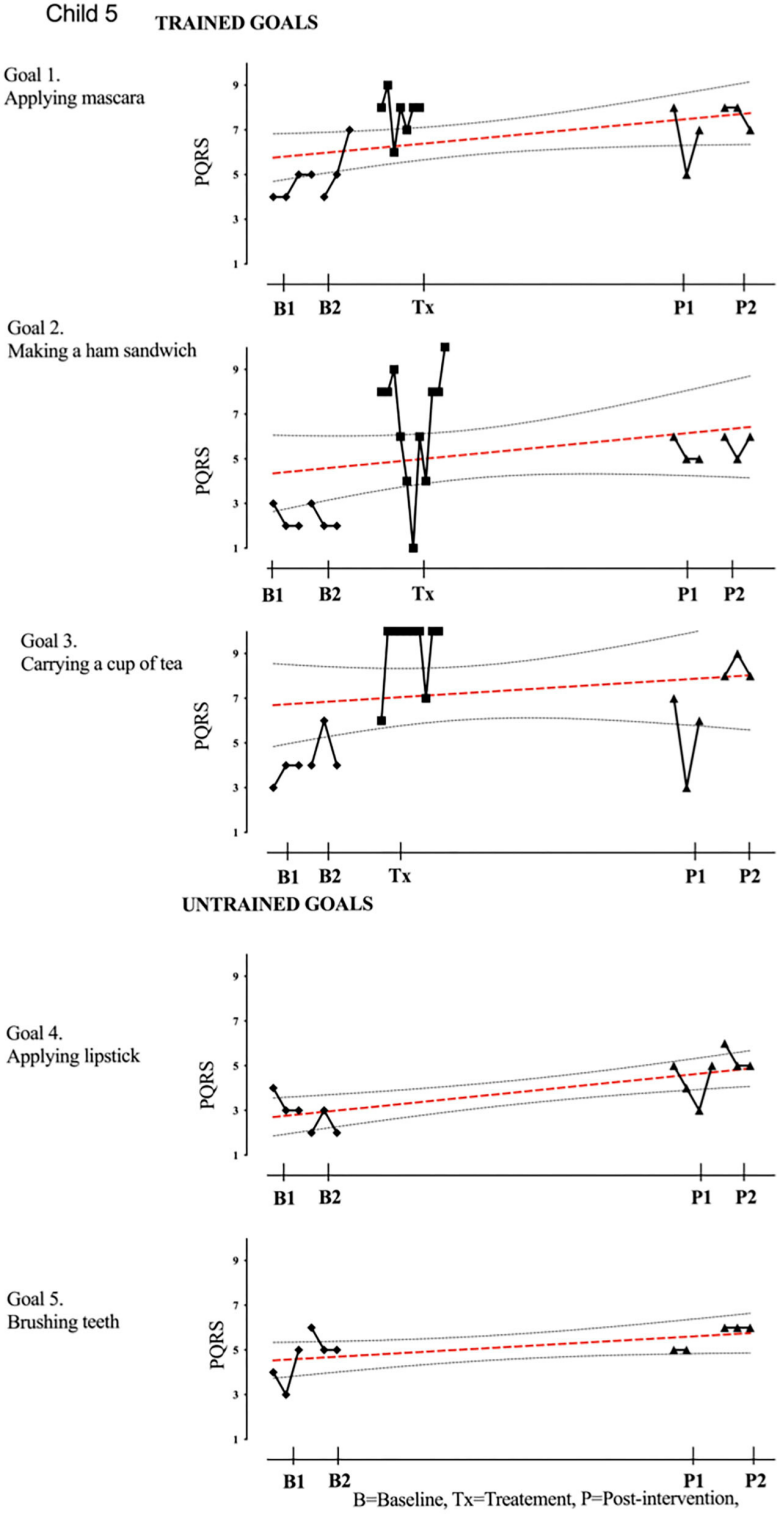
Child 5 PQRS-i scores (y-axis) for Gl-5 for each trial phase (x-axis). Results with OLS regression line superimposed.

**Figure 7:**
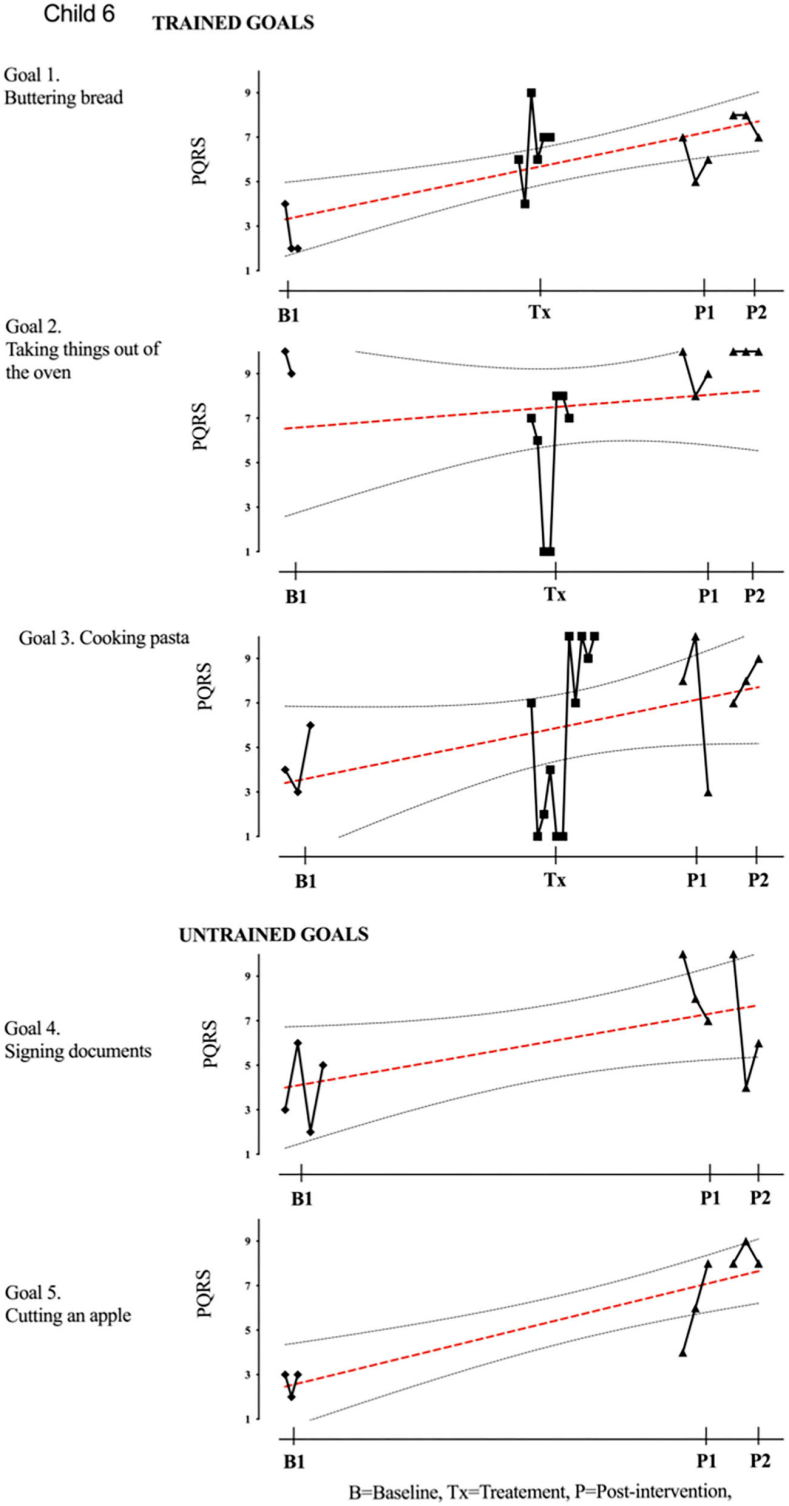
Child 6 PQRS-i scores (y-axis) for Gl-5 for each trial phase (x-axis). Results with OLS regression line superimposed.

**Figure 8:**
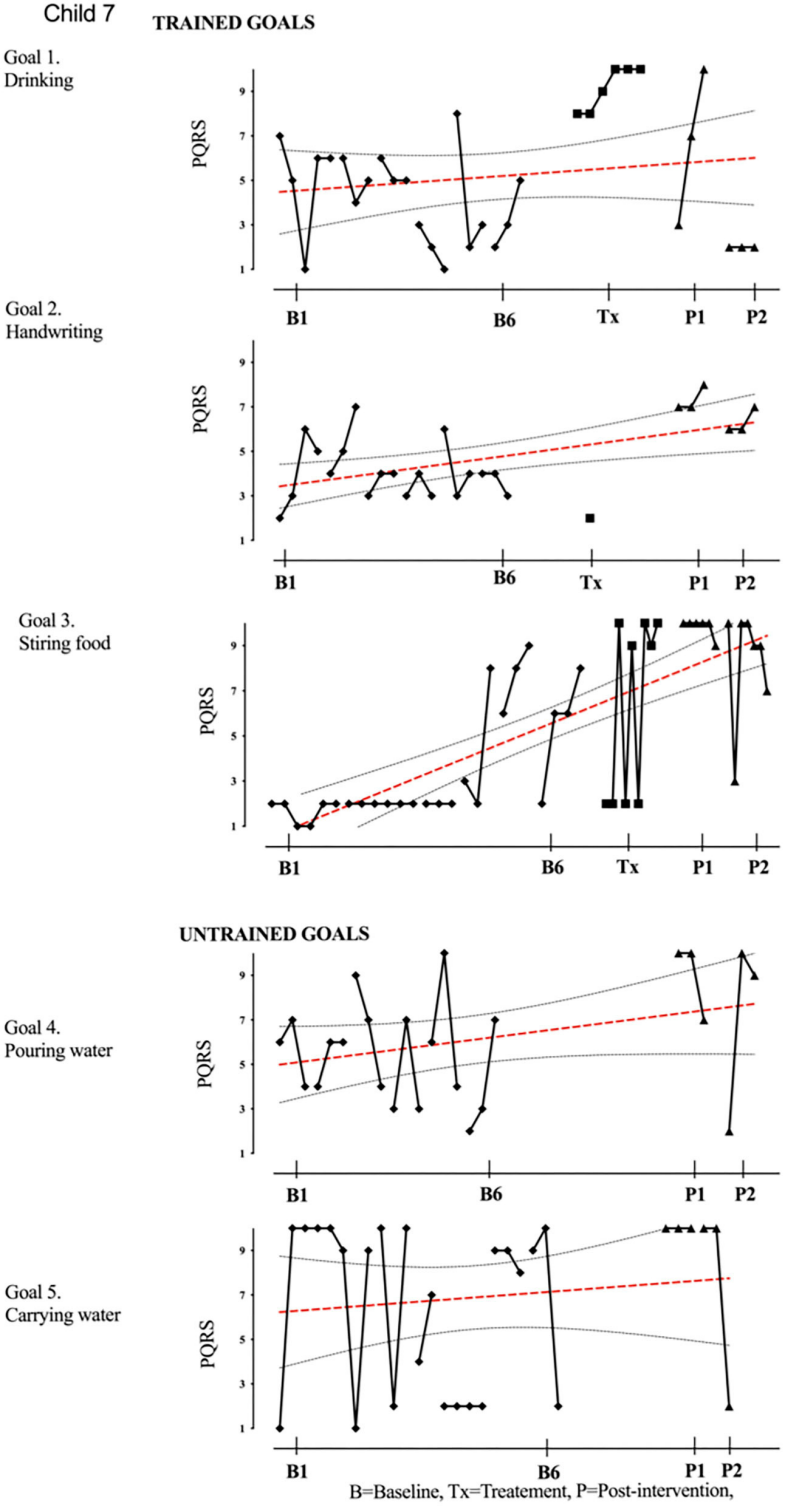
Child 7 PQRS-i scores (y-axis) for Gl-5 for each trial phase (x-axis). Results with OLS regression line superimposed.

**Figure 9:**
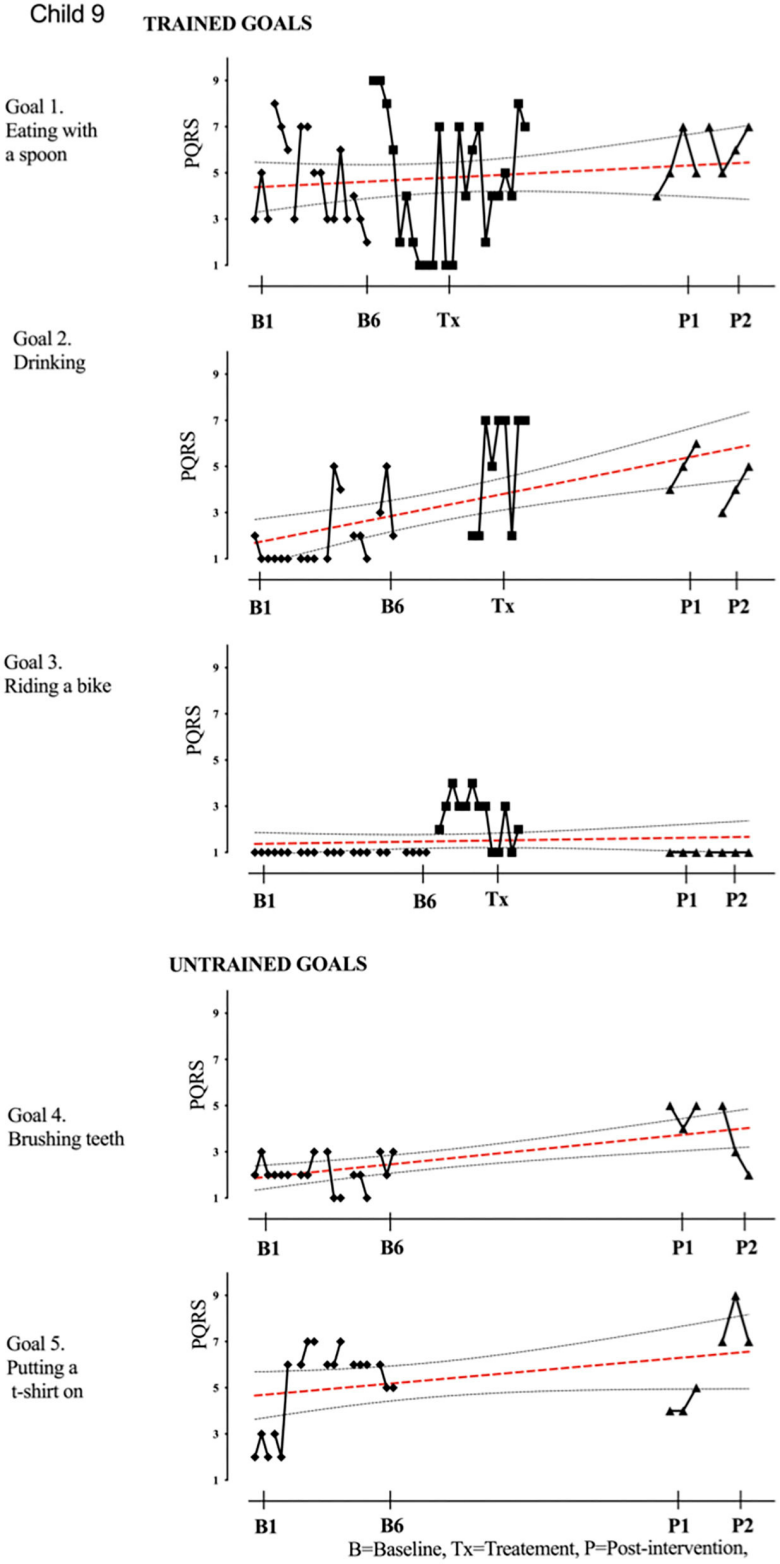
Child 9 PQRS-i scores (y-axis) for Gl-5 for each trial phase (x-axis). Results with OLS regression line superimposed.

**Figure 10:**
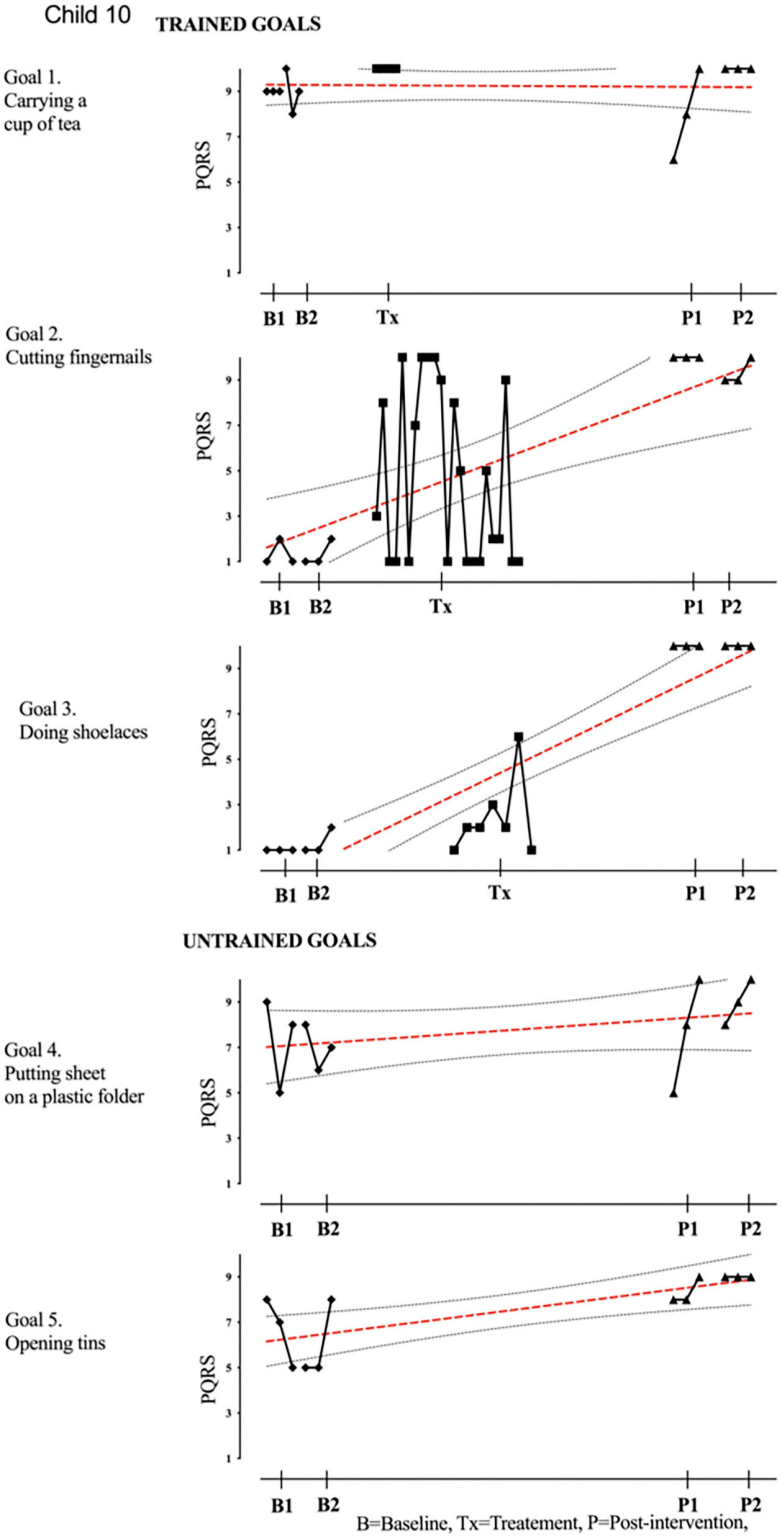
Child 10 PQRS-i scores (y-axis) for Gl-5 for each trial phase (x-axis). Results with OLS regression line superimposed.

**Figure 11:**
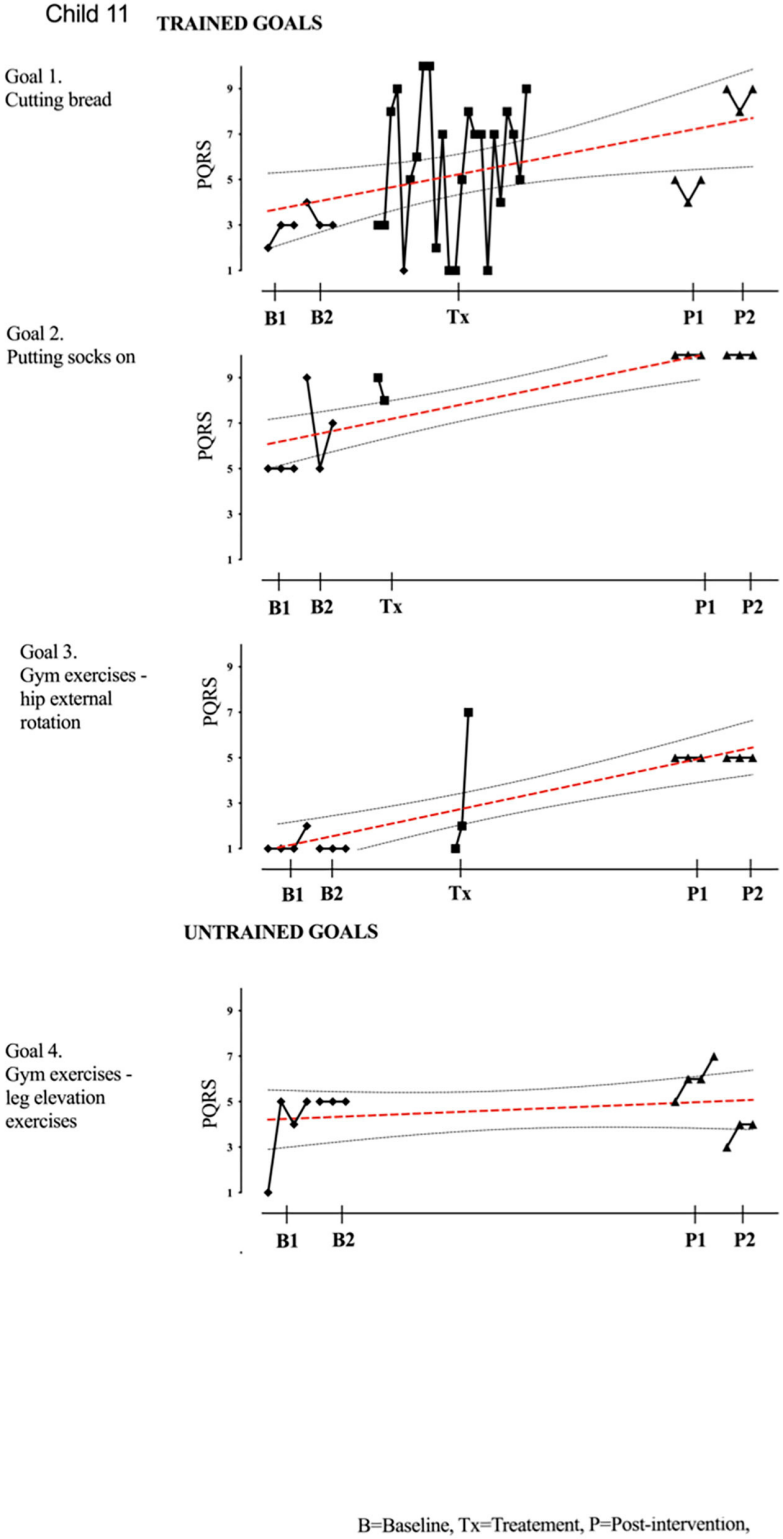
Child 11 PQRS-i scores (y-axis) for Gl-5 for each trial phase (x-axis). Results with OLS regression line superimposed.

**Figure 12:**
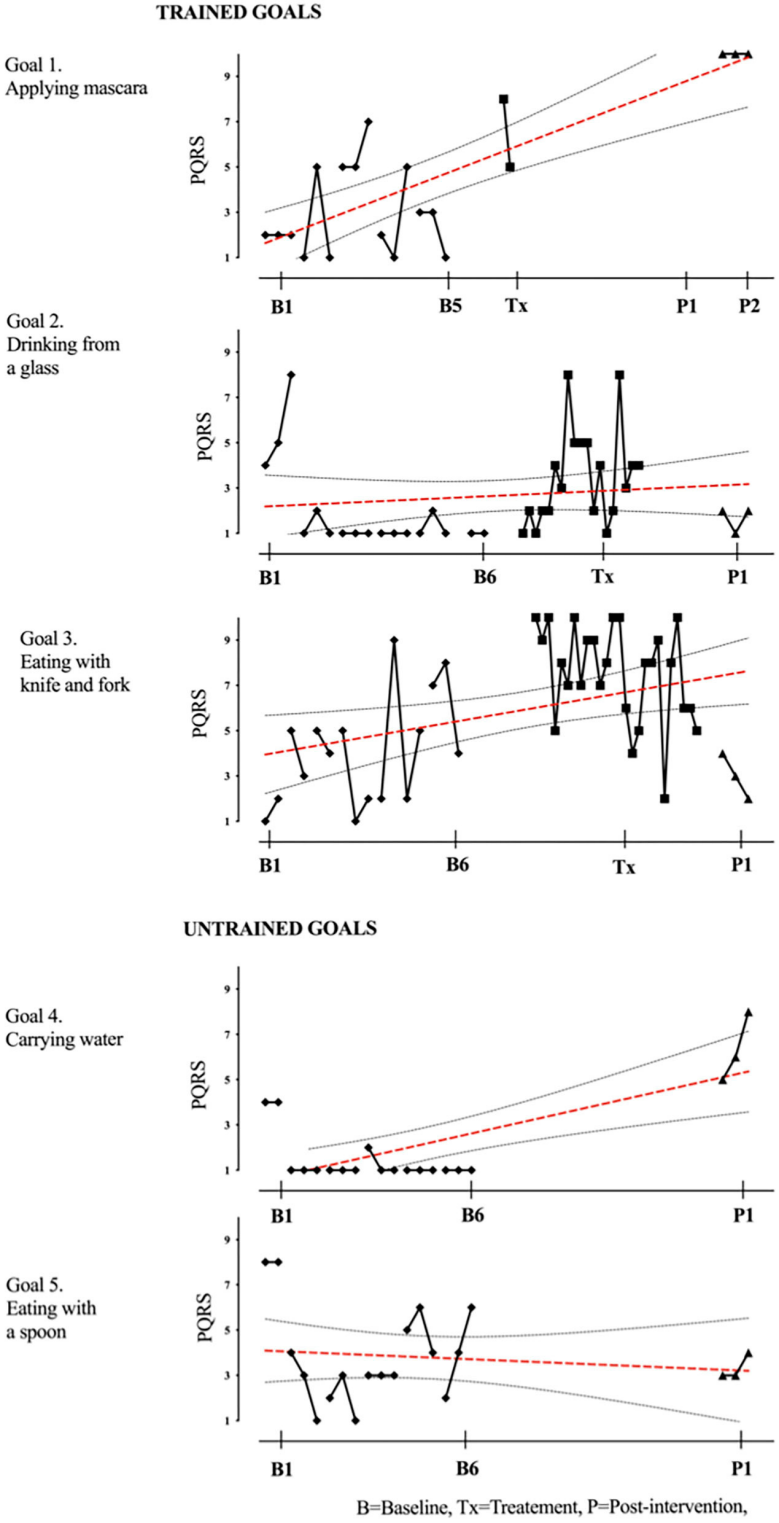
Child 12 PQRS-i scores (y-axis) for Gl-5 for each trial phase (x-axis). Results with OLS regression line superimposed.

Results of participant 1 are presented graphically with trained and untrained goals in Figure 2. The graph shows changes following intervention in all trained goals though mild in one of the three and significant in the other two goals. Figure 2 also shows transfer to untrained goals in one of the two goals chosen to measure this construct. The graphs for the remaining replication cases (including the case that did not proceed to intervention) are provided in Figures 3–12.

#### Quantitative Analysis of Performance Changes

Statistical differences between means in baseline and post-intervention supported the visual inspections and indicated that all participants achieved a significant improvement on at least one trained goal, with 63% (19/30) of goals improving across participants at post-test (Tables 2, 3). Two participants improved on all of their trained goals, five improved on two goals, and three improved on a single goal. For the untrained goals, 7 of the 10 participants receiving CO-OP improved on at least one of the untrained goals, with 37% (7/19) of goals improving overall. Two participants showed deterioration in one of the two selected untrained goals.

Baseline trend (i.e., auto-correlation) was found in six goals and therefore the use of Tau-*U* correction for baseline trend was used. Using this index, all participants achieved improvement in at least one goal with moderate or large effect size. A “large” effect (≥0.93) (“very effective intervention”) was obtained in 80% of children post-intervention. In total, the effect size was “large” for 37% of trained goals overall post-treatment. For other trained goals, “moderate” effects (0.66 to 0.92) (“effective intervention”) were obtained in 70% of children and for 27% of trained goals overall at post-intervention. “Weak” effects (≤0.65) were seen in 11 goals (37%) overall across eight children at post-intervention. No negative effects (deterioration) were observed for trained goals.

For untrained goals, “large” or “moderate” effect was seen in 37% of goals (7/19) overall in six children post-treatment, and “weak” effects in 53%. Deterioration with “moderate” negative effects were seen in two goals for two different children. Supplementary Information 2 summarizes results for effect size using non-overlapping index, Tau-*U*.

#### Clinically Significant Change

All participants showed a positive change of at least two PQRS-i points (based on the difference between the phase means) on at least one goal, at the end of treatment (Tables 2, 3). Post-intervention, 6 out of the 10 participants showed clinically significant improvement on two of their trained goals and two children improved on all trained goals. For untrained goals, six children showed significant transfer on at least one goal at post-intervention.

#### Quantitative Statistical Analysis of Performance Change Between DBS+CO-OP and DBS+Extended Baseline/Practice of the Goals)

Differences between means in extended baseline using *t*-test supported the visual inspections and indicated that DBS and practice alone did not offer improvement in the majority of goals (see Supplementary Information 3). There was, however, significant improvement in 4 out of the 25 goals for four out of five participants during extended baseline/practice. Significant deterioration was also noted for two participants for a total of three goals during extended baseline/practice.

#### Analysis of Results in Relation to Therapist-Related Fidelity to Treatment

The six treating therapists varied in years of experience from recently qualified to 20 years (see Supplementary Information 1). Two out of the six therapists achieved <50% on fidelity checklist with the lowest scores on two of the key elements of the CO-OP approach, Guided Discovery and collaborative Dynamic Performance Analysis (DPA). The two participants treated by the therapists with the lowest fidelity score achieved improvement in goals, but the majority of overall change was measured during the baseline phase with limited improvement over the course of therapy.

## Data Availability

Access to de-identified participant data may be requested by contacting the first author.

## Discussion

This is the second of two studies exploring the use of the CO-OP approach with childhood-onset HMD post-DBS. The first was set up as a proof of concept and preliminary efficacy (i.e., can the intervention be implemented?). The present study was set up as preliminary evaluation of effectiveness (i.e., can it be delivered in every day practice?) and reports the results in performance improvement when the intervention is delivered by local occupational therapists not specialized in HMD and with only basic training in the CO-OP approach based in centers across the UK. Together, the two studies provide evidence that CO-OP is a feasible and acceptable intervention for children and young people with HMD following DBS, with the potential to produce clinically meaningful improvement.

The results obtained in the present study are promising on two grounds: Firstly, although treatment fidelity was variable, CO-OP can be delivered by occupational therapists independent of their years of training and with a relative low-intensity training course, which makes this approach feasible in the context of clinical practice for further formal evaluation. Secondly, replicability has been demonstrated across multiple therapists, in a substantial number of children and young people with heterogeneous presentations of HMD and DBS duration. The total number of successful replications (*n* = 10) reported here and in combination with those reported previously (N-of-1 plus 8 replications) exceeds the three to five replications recommended in single-case experimental design, increasing confidence in the results. In this study, all eligible participants in the CMDS database were approached and all who consented were recruited to the study, reducing the risk of bias selection.

As in the first N-of-1 series with eight replications (7), skill improvement following CO-OP was seen in all children and young people, independently of their baseline characteristics and with a broad range of goals as outlined in Table 2. The majority (19/30) of goals addressed in therapy improved during the CO-OP intervention (63% goals improved compared to 75% of goals in the first study). This indicates slightly lower goal improvement rates than in the previous series completed by a therapist experienced in movement disorders and CO-OP. This is in line with reported literature indicating that effectiveness studies closer to the “real-world” setting often show lower improvement rates than in efficacy studies taking place in specialist centers (26). However, the results indicate that useful results are still possible with a relative brief therapy intervention (<10 h per participant) and low training requirements, as well as for therapists with no prior experience in HMD.

Results suggest that meaningful functional improvement is possible in children with a range of etiologies and clinical presentations in childhood-onset HMD. Half of the group achieved at least two out of the three goals worked in therapy. The participants achieving only one goal included one participant with childhood stroke, a participant with CP, and one participant with a metabolic disorder.

While robust positive change was observed on at least one trained goal by all participants, some goals showed no change. Of those children who improved on only one goal, the unimproved goals worked on were doing buttons and putting shoes on for the child with stroke (participant 4), toothbrushing and drinking for child with metabolic disorder (participant 9), eating with knife and fork, and applying mascara for child with CP (participant 12). Possible explanations for the lack of improvement could relate to the difficulty of the selected goal in relation the participants' motor impairment, the fidelity of treatment, or the expertise of the therapist. Although challenging, similar goals successfully improved in other participants with the same characteristics, suggesting that the goal itself may not be the limiting factor or the clinical characteristics. Therapist effects may have been important in some of the cases that showed limited clinical gains. Fidelity to treatment was rated <50% for case 4 (38%) and case 9 (49%) who both improved on only a single goal. Therefore, wider improvement might have been hindered by the diluted delivered version of the CO-OP intervention. It may well be that outcome relates to a combination of the difficulty in the chosen goal itself and the therapists' difficulty to apply the CO-OP approach to these more challenging goals. Future trials may require greater investment in training and supervision.

Although improvement on trained goals is an important outcome, the real potential of this treatment lies in its generalizability and transferability, enabling improving performance beyond the treatment sessions, thereby broadening the reach of the CO-OP approach. In the present study skill transfer was observed in seven of the untrained goals, across five participants. This is similar to the previous single-therapist HMD single-case experimental design (7) and in adults with stroke (27). Even if evidence for the generalizability of transfer is somewhat limited, the potential is demonstrated and warrants further work to enhance this crucial outcome.

Finally, the present study permitted an assessment of the impact of repeated practice on the goals with DBS *in situ* prior to the systematic application of the CO-OP approach. Improvement during extended baseline (DBS and practice) (Supplementary Information 3) was noted for 4 of 25 goals in four out of the five children randomly allocated to extended baseline. In such cases, it was hard to disentangle the effects of practice and neuromodulation with DBS. However, given that only some of the goals improved in the extended period (*n* = 4, 16%), it is more likely due to practice effects than DBS. In the remainder, who showed stable baseline performance, improvement could be more confidently attributed to the CO-OP approach.

As with any study, there are limitations that warrant mentioning. All of the participants in this study had DBS *in situ*, raising the possibility that DBS-related factors may moderate the outcome of CO-OP. The careful design of both, this and the former proof-of-concept study (7), allowed for manipulation of DBS length of neuromodulation as a variable. Those with neuromodulation in place for longer than 1 year, when most of the change has taken place, particularly in inherited genetic dystonias, showed improvement when the CO-OP intervention started and not necessarily on the baseline period. Secondly, the extended baseline used in this study allowed for close monitoring of change happening within a 6 week period with no therapy intervention, showing much less change on the goals set by the participants. Finally, and most importantly, given that neuromodulation is a global management approach, improvement would be expected to be seen across any goals (trained or untrained) the young person performs. This was not the case in either of the studies performed in the short study periods within these studies, indicating that transfer and indeed goal acquisition are most likely due to the effect of the CO-OP approach. However, goal attainments after DBS in genetic and acquired childhood onset dystonia disorders have been reported without CO-OP at 1–2 years post-DBS implant (28). Without DBS intervention, dystonia worsens (29) along with fixed deformities (30) irrespective of the cause and conventional surgical and medical approaches failed to address the needs of children with HMD (31). DBS neuromodulation is a global management approach to reducing dystonia, chorea, myoclonus, and tremor, often in children with little or no pre-existing motor repertoire in whom dystonia reduction does not equate with spontaneous acquisition of skills after DBS intervention, even with conventional practice and repetition of desired motor skill. For the networked efficacy of DBS, motor and sensory pathways must be intact (32). In addition, cognitive function appears preserved or even enhanced following DBS in isolated-genetic (33), and in addition to cognitive stability, perceptual reasoning may be increased after DBS in acquired (34) dystonias, respectively, supporting the place of cognitive strategies to boost goal acquisition and transfer of skills to untrained goals with CO-OP approach after DBS.

It is therefore postulated that DBS accelerates or facilitates the efficacy of CO-OP by reducing dystonia and also by modifying the underlying cerebral plasticity. Since, as a guide, it may take up to 2 years for the full benefits of DBS to manifest in isolated genetic dystonias and longer in acquired dystonias, applying methods that enhance the overall speed of goal attainment through a cognitive problem-solving approach is clearly urgently required in childhood when the windows of plasticity are limited (35).

In conclusion, the presented results are promising for a number of reasons. This trial is the first attempt to systematically evaluate the potential effectiveness of a rehabilitation intervention for children and young people with HMD across therapists. Although further testing of efficacy and effectiveness through large-scale trials is required, the present study shows that CO-OP is a feasible and acceptable approach to rehabilitation following DBS for children and young people with HMD and that changes are overall significant for client-chosen goals, including in children with dyskinetic CP, for whom DBS or any other current management modality does not currently provide enough functional changes.

As described, fidelity to treatment was variable and sometimes sub-optimal, indicating the importance of training and supervision in future trials. The limited transfer achieved to untrained goals also warrants further investigation in relation to delivery of the intervention, dosage (timing, frequency, and duration), and any modifications that might be required to achieve transfer to goals not addressed in therapy for this population. Finally, the promising results from using CO-OP with HMD and DBS warrant further investigation of the CO-OP approach in dystonia, which offers a treatment option for a wider number of potential patients. Further, the results from this study and the previous study indicate that practice alone does not provide improvement in self-selected goals. This suggest that simple goal-oriented approaches recommended by experts in dystonia might not be sufficient without the more cognitive problem-solving element inherent in CO-OP. Future research comparing CO-OP with other goal-oriented approaches would be valuable.

## Data Availability Statement

The raw data supporting the conclusions of this article will be made available by the authors, without undue reservation.

## Ethics Statement

The studies involving human participants were reviewed and approved by Oxford A Research Ethics Committee, 14/SC/1159. Written informed consent to participate in this study was provided by the participants' legal guardian/next of kin.

## Author Contributions

HG completed all statistical analysis. This was supervised by VC, senior trial statistician. HG: research study, conception, organization, execution, manuscript preparation, and writing of the first draft. HP, J-PL, VC, and RB: research study, conception, manuscript preparation, and review and critique. All authors contributed to the article and approved the submitted version.

## Conflict of Interest

J-PL has received unrestricted educational support for instructional courses and consultancy fees from Medtronic Ltd. The remaining authors declare that the research was conducted in the absence of any commercial or financial relationships that could be construed as a potential conflict of interest.
