# Supplementary material for: Cognitive Strategy Training in Childhood-Onset Movement Disorders: Replication Across Therapists
Source: Front Pediatr. 2021 Jan 21;8:600337. doi: 10.3389/fped.2020.600337 (PMC7861040; doi:10.3389/fped.2020.600337)
Supplement: Supplementary file 1 [file Table_1.docx]

Supplementary Information-1. Therapist’s characteristics (n=6)

| **Therapist** | **Years of experience** | **Training attended** | **% Fidelity to Treatment** | **Children treated** |
| --- | --- | --- | --- | --- |
| 1 | 5 years | CO-OP 3 day UK | 82% | Participant 1 |
| 2 | 4 years | CO-OP 2 day UK | 75% | Participant 2 |
| 3 | 20 years | CO-OP 3 DAY UK | 38% | Participant 4 |
| 4 | Newly qualified | CO-OP 2 day non-UK | 82% | Participants 5, 8, 10, 11, 12 |
| 5 | 20 years | CO-OP 3 DAY UK | 91% | Participant 6 |
| 6 | 9 years | CO-OP 2 day non-UK | 49% | Participant 9 |
