# Supplementary material for: Cognitive Strategy Training in Childhood-Onset Movement Disorders: Replication Across Therapists
Source: Front Pediatr. 2021 Jan 21;8:600337. doi: 10.3389/fped.2020.600337 (PMC7861040; doi:10.3389/fped.2020.600337)
Supplement: Supplementary file 2 [file Table_2.docx]

Supplementary Information SI-2. Effect size calculation per goal and per participant using Tau-*U* values, p values and 90% confidence intervals

| **Subject (GT)** | **Tau-*U* Baseline to post** | | | | **Subject (GT)** | **Tau-*U* Baseline to post** | | |
| --- | --- | --- | --- | --- | --- | --- | --- | --- |
|  | **Tau-*U* value** | | **p value (90% CI)** | **Effect size** |  | **Tau-*U* value** | **p value (90% CI)** | **Effect size** |
| **TRAINED GOALS** | | | | | **UNTRAINED GOALS** | | | |
| 1(1) | 0·56 | 0·11 (-0·02<>1) | | Weak | 1(4) | 0·9 | 0·003 (0·39<>1) | Strong |
| 1(2) | 0·67 | 0·06 (0·10<>1) | | Moderate | 1(5) | 0·42 | 0·23 (0·15<>0·99) | Weak |
| 1(3) | 1 | 0·004 (0·43<>1) | | Strong |  |  |  |  |
| 2(1) | -0·43 | 0·24 (-1<>0·17) | | Weak | 2(4) | -0·75 | 0·031 (-1<>-0·18) | Moderate worse |
| 2(2) | 1 | 0·02 (0·29<>1) | | Strong | 2(5) | 0 | 1 (-0·71<>0·71) | Weak |
| 2(3) | 0.83 | 0·016 (0.26<>)1 | | Moderate |  |  |  |  |
| 4(1) | 0·74 | 0·003 (0·32<>1) | | Moderate | 4(4) | 0·28 | 0·34 (-0·20<>0·77) | Weak |
| 4(2) | 0·50 | 0·10 (0·00<>0·99) | | Weak | 4(5) | -0·75 | 0·03 (-1<>-0·19) | Moderate worse |
| 4(3) | 0·66 | 0·03 (0.15<>1) | | Moderate |  |  |  |  |
| 5(1) | 0·83 | 0·01 (0·29<>1) | | Moderate | 5(4) | 0·86 | 0·01 (0·31<>1) | Moderate |
| 5(2) | 1 | 0·003 (0·43<>1) | | Strong | 5(5) | 0·57 | 0·12 (-0·03<>1) | Weak |
| 5(3) | 0·69 | 0·034 (0·14<>1) | | Moderate |  |  |  |  |
| 6(1) | 1 | 0·02 (0·29<>1) | | Strong | 6(4) | 0·79 | 0·04 (0·15<>1) | Moderate |
| 6(2) | 0·17 | 0·74 (-0·66<>0·99) | | Weak | 6(5) | 1 | 0·02 (0·29<>1) | Strong |
| 6(3) | 0·72 | 0·09 (0·01<>1) | | Moderate |  |  |  |  |
| 8(1) | 0·4 | 0·14 (-0·05<>0·85) | | Weak | 8(4) | 0·55 | 0·05 (0·09<>1) | Weak |
| 8(2) | 0·90 | 0·001 (0·45<>1) | | Moderate | 8(5) | 0·49 | 0·07 (0·04<>0·92) | Weak |
| 8(3) | 0·92 | 0 (0·59<>1) | | Strong |  |  |  |  |
| 9(1) | 0·38 | 0·13 (-0·03<>0·79) | | Weak | 9(4) | 0·77 | 0·006 (0·31<>1) | Moderate |
| 9(2) | 0·81 | 0·004 (0·35<>1) | | Moderate | 9(5) | 0·24 | 0·39 (-0·21<>0·70) | Weak |
| 9(3) | 0 | 1(-0·42<>0·42) | | Weak |  |  |  |  |
| 10(1) | 0·25 | 0·47 (-0·32<>0·82) | | Weak | 10(4) | 0·44 | 0·20 (-0·13<>1) | Weak |
| 10(2) | 1 | 0·004 (0·43<>1) | | Strong | 10(5) | 0·89 | 0·01 (0·32<>1) | Moderate |
| 10(3) | 1 | 0·004 (0·43<>1) | | Strong |  |  |  |  |
| 11(1) | 0·97 | 0·005 (0·40<>1) | | Strong | 11(4) | 0·02 | 0·95 (-0·51<>0·55) | Weak |
| 11(2) | 1 | 0·004 (0·43<>1) | | Strong | 11(5) | Not set | Not set | Not set |
| 11(3) | 1 | 0·003 (0·45<>1) | | Strong |  |  |  |  |
| 12(1) | 1 | 0·025 (0·26<>1) | | Strong | 12(4) | 1 | 0·007 (0·39<>1) | Strong |
| 12(2) | 0·22 | 0·55 (-0·38<>0·83) | | Weak | 12(5) | -0·08 | 0·83 (-0·69<>0·53) | Weak |
| 12(3) | -0·22 | 0·54 (0·84<>0·38) | | Weak |  |  |  |  |

Abbreviations: GT: Goal trained, GUT: goal untrained, SD: Standard Deviation, CI: Confidence Interval, NT: non-tested.

Table 4 presents effect size calculations per goal and per participant using Tau-*U* values, p values and 90% confidence intervals. Effect size is presented as weak, moderate, and strong (including those with negative trend). The majority of participants achieve moderate and strong effect size in their chosen trained goals, to a lesser degree this is a similar pattern for the untrained goals. Finally, two goals for two separate participants show effect size moderately worse.
