# Supplementary material for: Cognitive Strategy Training in Childhood-Onset Movement Disorders: Replication Across Therapists
Source: Front Pediatr. 2021 Jan 21;8:600337. doi: 10.3389/fped.2020.600337 (PMC7861040; doi:10.3389/fped.2020.600337)
Supplement: Supplementary file 3 [file Table_3.docx]

Supplementary Information SI-3. Results of t-test analysis for extended baseline with negative T-test indicating improvement in change Pre_1_-Pre_6_ whilst positive t-test scores indicate negative trend

| **Child number (goal)** | **Improvement** | **T-test Pre_1_-Pre_6_** | |  | |  | | **Improvement** | **T-test Pre_1_-Pre_6_** |  |  |
| --- | --- | --- | --- | --- | --- | --- | --- | --- | --- | --- | --- |
|  |  | Mean shift (95%CI) | | P | |  | |  | Mean shift (95%CI) | P |  |
| **TRAINED GOALS** | | | | | | | **UNTRAINED GOALS** | | | | |
| 3(1)** | Deterioration | 1·60 (0·51, 2·69) | 0·012 | | 3(4) | | | No change | -0·90 (-2·46, 0·66) | 0·225 |  |
| 3(2) | No change | -0·33 (-1·44, 0·78) | 0·450 | | 3(5)** | | | Improvement | -1·67 (-3·33, -0·004) | 0·050 |  |
| 3(3) | No change | 0·33 (-1·15, 1·81) | 0·637 | |  | | |  |  |  |  |
| 4(1) | No change | -0·08 (-0·27, 0·10) | 0·339 | | 4(4) | | | No change | -0·40 (-11·07, 10·27) | 0·912 |  |
| 4(2) | No change | 0·15 (-2·05, 2·35) | 0·868 | | 4(5) | | | No change | 0·50 (-2·62, 3·62) | 0·707 |  |
| 4(3) |  | No change |  | |  | | |  |  |  |  |
| 8(1) | No change | 0·49 (-6·30, 7·28) | 0·813 | | 8(4) | | | No change | 0·25 (-2·66, 3·16) | 0·837 |  |
| 8(2) | No change | -0·08 (-4·98, 4·81) | 0·952 | | 8(5) | | | No change | 1·13 (-10·45, 12·71) | 0·748 |  |
| 8(3)*** | Improvement | -2·44 (-3·97, -0·92) | 0·004 | |  | | |  |  |  |  |
| 9(1) | No change | -0·58 (-2·78, 1·61) | 0·516 | | 9(4) | | | No change | 0·25 (-0·86, 1·36) | 0·569 |  |
| 9(2) | No change | -1·00 (-2·25, 0·25) | 0·104 | | 9(5)** | | | Improvement | -3·75 (-4·88, -2·62) | 0·001 |  |
| 9(3) |  | No change |  | |  | | |  |  |  |  |
| 12(1) | No change | -1·56 (-3·15, 0·03) | 0·054 | | 12(4)*** | | | Deterioration | 2·91 (2·71, 3·11) | <0·001 |  |
| 12(2) | No change | 4·50 (-0·61, 9·61) | 0·063 | | 12(5)** | | | Deterioration | 4·36 (3·31, 5·41) | <0·001 |  |
| 12(3)*** | Improvement | -3·00 (-5·34, -0·66) | 0·018 | |  | | |  |  |  |  |
